# Prevalence and Contributing Factors of Occupational Injuries Among Nurses in Oshakati District, Namibia

**DOI:** 10.3390/ijerph22060912

**Published:** 2025-06-08

**Authors:** Maliwa Lichaha Sanjobo, Mpinane Flory Senekane, Kgomotso Lebelo

**Affiliations:** 1 Department of Environmental Health, Faculty of Health Sciences, University of Johannesburg, Johannesburg 2094, South Africa; sanjoboml@gmail.com (M.L.S.); msenekane@uj.ac.za (M.F.S.); 2 Occupational Health Division, School of Public Health, Faculty of Health Sciences, University of the Witwatersrand, Johannesburg 2193, South Africa

**Keywords:** occupational injuries, prevalence, nurses, health workers, Namibia

## Abstract

Occupational injuries among nurses impact their well-being and health service delivery. This study aimed to determine the prevalence of occupational injuries and their contributing factors among nurses in selected public health facilities. A descriptive cross-sectional study was conducted among 295 randomly selected nurses. Primary data were collected using self-administered questionnaires, while secondary data were abstracted from the occupational injuries register. Data were analysed using statistical software. The 12-month prevalence of occupational injuries was 28.8% (95% confidence interval [CI] = 24.3–33.3%), with needlestick injuries being the most common (63.5%). Significant associations were observed between occupational injuries and education level (*p* = 0.027), employment status (*p* = 0.012), and years of experience (*p* = 0.029). Nurses with a bachelor’s degree had 3.30 times higher odds of injury (Adjusted OR [AOR] = 3.30, 95% CI = 1.11–9.81, *p* = 0.03), while the lack of proper training increased the odds by 3.27 times (AOR = 3.27, 95% CI = 1.62–6.61, *p* < 0.001). Being a registered nurse reduced the odds by 70% (AOR = 0.30, 95% CI = 0.12–0.74, *p* = 0.01). Addressing these factors is essential for reducing occupational injuries and improving healthcare outcomes.

## 1. Introduction

Occupational injuries remain a significant global public health concern, affecting millions of workers annually. According to the United Nations Global Compact [1], approximately 374 million workers experience non-fatal occupational accidents each year. Healthcare workers are particularly vulnerable, with over 2 million sustaining needlestick injuries (NSIs) and 63% exposed to workplace violence annually [2]. For this study, an occupational injury refers to trauma inflicted on body tissue as a result of an accidental event occurring during work, leading to personal harm for one or more workers [3]. The classification of injuries, based on the International Statistical Classification of Diseases and Related Health Problems (ICD 11), includes categories such as superficial injuries, open wounds, fractures, dislocations, sprains, strains, burns, corrosions, scalds, and frostbite [4].

Globally, emerging trends indicate a rising incidence of occupational injuries among health workers, driven by factors such as excessive workloads, shift patterns, and mental health stressors [5,6]. Reported injury rates range from 29.7% to 60.17%, with NSIs, falls, and workplace violence being the most common incidents [7,8]. Underreporting remains a persistent issue, often linked to a lack of knowledge about reporting procedures or fear of blame [9]. The severity of occupational injuries varies, influenced by factors like occupational safety policies, workplace environments, industry characteristics, and socioeconomic conditions. Injury severity can be assessed by examining the frequency, types, and overall impact on employees, workplaces, and broader economies [4]. Unsafe working environments that contribute to occupational injuries and worker absenteeism can impose a significant financial cost of up to 2% of global health spending [2]. Occupational injury rates remain high, especially in low- and middle-income countries, where resource constraints, outdated guidelines, and limited training exacerbate injury risks [10,11]. In sub-Saharan Africa, healthcare systems often struggle with resource limitations, understaffing, and inadequate safety equipment, which increase injury risks [12].

Nurses comprise the largest segment of the global health workforce, accounting for approximately 59% of healthcare professionals [13]. They are vital in delivering patient care, often in demanding and high-pressure environments. As healthcare providers who spend the most time in direct contact with patients across various healthcare settings, they continuously contribute to patient well-being and the efficient operation of health systems [14]. Unfortunately, this commitment exposes them to a heightened risk of occupational injuries, jeopardising their health and safety [10].

Nurses face multiple hazards in their work environments, including physical, chemical, biological, and ergonomic risks. Such hazards can result in injuries that compromise their well-being and affect the quality of care they provide [15]. The nature of their work makes them vulnerable to various injuries, such as NSIs, strains and sprains from slips or falls, electric shocks, ambulance-related accidents, and even physical violence from patients, their families, or colleagues [2]. This subsequently exposes them to infectious diseases like Hepatitis B, Hepatitis C, and HIV [13,16]. The impact of these injuries extends beyond physical harm, affecting nurses’ mental well-being, leading to increased absenteeism, emotional distress, decreased productivity, compromised care quality, and higher attrition rates within the nursing profession [17]. Safeguarding nurses’ well-being is essential for their health and safety and sustaining high-quality patient care and strengthening healthcare systems globally.

In Namibia, nurses often work under stressful conditions, leading to potential injury risks [18]. In the Namibian context, nurses are either registered or enrolled. Registered nurses complete more extensive training, often at a diploma or degree level, and are authorised to perform a wider range of clinical and managerial duties independently. In contrast, enrolled nurses typically undergo a shorter period of training and have a more limited scope of practice, often focused on basic nursing care under supervision.

Despite progress in healthcare systems in Namibia, many facilities grapple with staff shortages, limited resources, and inadequate infrastructure [19]. These persistent issues significantly strain nurses, who frequently endure long working hours and stressful conditions [20]. Studies indicate that nurses in Oshakati’s health facilities frequently experience NSIs, musculoskeletal injuries, and physical assaults [7]. A study in Namibia [7] reported the prevalence of NSIs at 37.5%. These injuries not only impair nurses’ ability to provide care but also place additional strain on already stretched healthcare services. Addressing these challenges requires a multifaceted approach, including policy reforms, workplace culture improvements, comprehensive training programs, and better access to safety equipment [11]. Despite the evident burden of occupational injuries among nurses, research on this issue remains limited in Namibia [21], particularly in the Oshakati District.

This study aimed to determine and describe the 12-month prevalence of occupational injuries among nurses in the Oshakati District, as well as the factors contributing to these injuries in selected public health facilities. Understanding the extent and root causes of these injuries is crucial for informing policy reforms, safety training initiatives, and allocating resources to enhance workplace safety and healthcare service quality [14]. This study hypothesised that the prevalence of occupational injuries among nurses in selected public health facilities in Oshakati District, Namibia, is influenced by various contributing factors such as age, gender, years of experience, workload, shift patterns, availability of safety equipment, training, and work environment conditions, which play a role in determining the frequency and nature of injuries experienced by nurses in their workplace.

## 2. Materials and Methods

### 2.1. Study Design

A cross-sectional study design provided a current snapshot of occupational injuries. The survey included a mono-method approach, focusing on quantitative, descriptive research. The positivist approach, which relies on objective observation and direct experience [22], supported statistical analyses. This study recorded injuries over the past year to account for seasonal variations and fluctuations in healthcare demand.

### 2.2. Study Site

**Figure 1:**
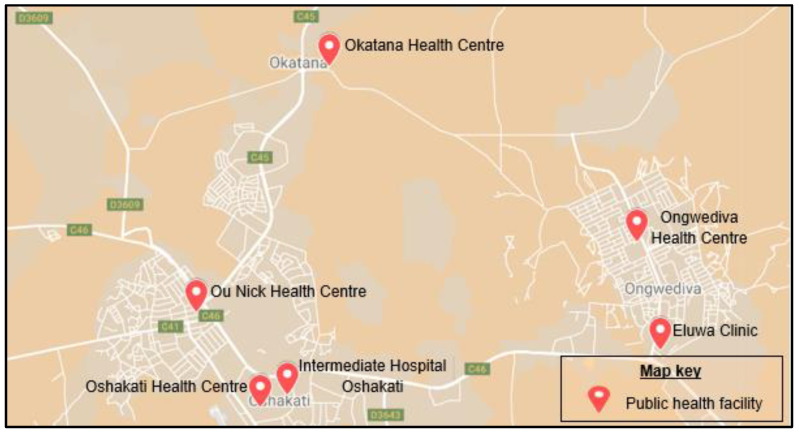
Public health facilities in Oshakati District [25].

This research was conducted in the Oshakati District, Oshana region, a key healthcare hub in Northern Namibia with a population of 33,618 [23]. The region covers 8653 km², with a population density of 20.43/km² [23]. This study focused on public health facilities under the Ministry of Health and Social Services (MoHSS): Intermediate Hospital Oshakati, Ou-nick Health Centre, and Oshakati Health Centre (Figure 1). Intermediate Hospital Oshakati, with 750 beds, treats nearly a million patients annually and serves as both a referral and training hospital [20,24]. Intermediate Hospital Oshakati, although located in a relatively small geographic area, functions as a referral hospital for Namibia’s entire northern region, receiving patients from multiple surrounding districts. Therefore, while the immediate land area and population size of Oshakati District may seem limited, the actual catchment population served by the hospital is significantly larger. Ou-nick and Oshakati Health Centres provide accessible primary healthcare services, strengthening Oshakati’s urban healthcare infrastructure.

### 2.3. Study Population

This study included registered and enrolled nurses actively working in the selected public health facilities. Intermediate Hospital Oshakati employed 612 nursing staff—323 registered and 289 enrolled [26]. Both nurse categories accounted for varied roles, responsibilities, and risk exposures.

### 2.4. Sampling Strategy

Proportional stratified random sampling ensured accuracy and generalisability. Nurses from selected public health facilities were categorised into strata based on their department (such as maternity, outpatient, casualty, surgical, and paediatric), type (registered or enrolled), and health facility (Intermediate Hospital Oshakati, Oshakati, and Ou-Nick health centres). Detailed lists formed the sampling frame, with participants randomly selected within each stratum using the Random Integer Generator (Random.org, version 2.0, Dublin, Ireland) to minimise selection bias. Questionnaires were distributed in each department according to its sample size, calculated using the following formula: (sample size/population size) × stratum size.

Questionnaires were distributed according to each department’s sample size. Inclusion criteria were all registered or enrolled nurses employed for over a year and validly registered with the Nursing Council of Namibia. Exclusion criteria included nurses employed for less than a year and those unable to provide informed consent.

### 2.5. Sample Size Estimation

The sample size was calculated using Epi Info version 7.2.6.0, developed by the Centers for Disease Control and Prevention (CDC), Atlanta, GA, USA. A 95% confidence interval and 80% power level were applied. A design effect of 1 and a 5% margin of error were used. Based on an expected injury prevalence of 50%, the calculated sample size was 236. A 50% prevalence rate is a standard and conservative estimate often used in cross-sectional studies when the actual prevalence is unknown or no prior data are available. It maximises the required sample size, ensuring sufficient statistical power to detect associations between variables. A 25% contingency (59 participants) was added to account for attrition, resulting in a final sample size of 295 participants.

### 2.6. Ethical Considerations

Ethical approval to conduct this study was obtained from the University of Johannesburg’s Research Ethics Committee (REC-3063-2024). Local permission was obtained from the Executive Director in the Ministry of Health and Social Services, and subsequently from the Intermediate Hospital Oshakati and the Oshakati Health District Office in Namibia. The conditions set by the ethics committees were reviewed and strictly adhered to. Participants were approached in their workplaces and informed about this study’s purpose and significance. Detailed information sheets explaining this study’s objectives, procedures, risks, and benefits were provided to all participating nurses. Informed written consent was obtained from respondents before participation in the study.

### 2.7. Data Collection and Management

A pilot study with 10 nurses was conducted at Ongwediva Health Centre, a health facility within the study area. Feedback was used to refine the data collection instruments. This study employed a survey and registry-based approach to collect data from primary and secondary sources. Data collection occurred from 22 October to 20 November 2024. Primary data were collected using structured, self-developed, self-administered, pre-coded questionnaires to gather quantitative data. The questionnaire aligned with the study objectives and included Likert scale ratings, closed-ended questions, and statements. Questionnaire sections covered demographics, occupational injury experiences, and contributing factors. Secondary data were abstracted from occupational injury registers maintained by the District Infection Control Department within the Intermediate Hospital Oshakati, using a pre-designed, self-developed abstraction form. Sections in the abstraction form included demographic data, injury experiences, and contributing factors. Secondary data served as a supplementary reference to confirm trends observed in the questionnaire responses.

Face validity was confirmed through simple and clear language in the questionnaire and participant feedback on the clarity and relevance of questions. Content validity was maintained by aligning questions with the study objectives and the literature. This study’s reliability was confirmed by a Cronbach’s alpha value of 0.79, indicating an acceptable level of internal consistency. In addition, reliability was upheld through pilot testing, the comprehensive training of data collectors, standardised data collection procedures, double-entry techniques, and strict compliance with approved protocols.

Data collection was scheduled for less demanding periods, such as night shifts and weekends, to align with nurses’ availability. Many healthcare facilities did not maintain occupational injury registers, requiring reliance on data from the District Infection Control Department in the Oshakati Intermediate Hospital. Daily checks were implemented to identify and rectify missing, duplicate, or inconsistent responses to uphold data quality. A double-entry method was used to reduce the risk of data entry errors. Participant information was kept confidential and not disclosed to parties outside the research team. Digital data was encrypted using Advanced Encryption Standard (AES)-256, with access to passwords and encryption keys restricted to the research team. The data were stored on OneDrive and backed up on a weekly basis. The research team securely managed all collected data and data collection tools at all times.

### 2.8. Data Analysis

The collected data were thoroughly checked and systematically edited to ensure accuracy, completeness, and internal consistency. Initially, the data were entered into Microsoft Excel for preliminary organisation before being exported to IBM SPSS Statistics for Windows, version 29.0., Armonk, NY, USA: IBM Corporation, for advanced statistical processing. The data-cleaning process involved detecting and addressing inaccuracies, omissions, duplications, and formatting inconsistencies to enhance data reliability and integrity. Missing values were systematically identified through frequency analyses and thereafter corrected. Standard scores (Z-scores) were used to flag potential out-of-range values for further verification. Outlier detection was conducted using descriptive statistical methods, with flagged values cross-checked against original records to confirm their validity and ensure data quality.

Descriptive statistics, including frequencies, percentages, means, standard deviations, medians, and interquartile ranges, were used to summarise demographic characteristics and key study variables, providing a comprehensive dataset overview. The Kolmogorov–Smirnov test assessed data normality, guiding the selection of appropriate parametric or non-parametric statistical tests [27]. Categorical data were presented using bar graphs, pie charts, and tables for a more straightforward interpretation and comparison of distributions. Inferential statistics were applied to examine associations between categorical variables, including chi-square tests and confidence intervals. A significance level of *p* ≤ 0.05 was used to determine statistical significance. Univariate and multivariate logistic regression analyses were conducted to assess the associations between dependent and independent variables, with multivariate regression controlling for potential confounders to enhance the reliability of findings.

## 3. Results

### 3.1. Demographic Characteristics for Primary Data

A total of two-hundred and ninety-five (295) questionnaires were completed, yielding a 100% response rate. In addition, seven records were abstracted from the occupational injury register maintained by the District Infection Control Department. Most participants were female (84.7%, n = 250), which aligns with the well-documented predominance of women in the nursing workforce. Regarding age distribution, the most significant proportion of respondents were aged 26–35 (43.4%, n = 128), followed by those aged 36–45 (28.5%, n = 84). The mean age of participants was 35.68 years (SD = 9.95), indicating moderate variability in age distribution. The median age was 34.5 years, with an interquartile range (IQR) of 30.5–40.5 years, suggesting that the middle 50% of participants fell within this range. The mean age is higher than the median, indicating a positively skewed distribution [28], likely influenced by a small number of older participants. Regarding educational qualifications, nearly half of the respondents (49.8%) held a certificate or diploma, while 41.7% had a bachelor’s degree.

The distribution of the respondents was across various wards, with the highest representation in the maternity ward (20.7%, n = 61), followed by the surgical ward (10.2%, n = 30) and then the outpatient department (9.8%, n = 29). Fewer participants worked in specialised areas, such as the intensive care unit (ICU; 3.4%, n = 10) and oncology (2.0%, n = 6). When comparing the number of respondents between hospital departments and the two health centres, most participants (87.1%, n = 257) worked in hospital departments. Oshakati Health Centre had 9.2% (n = 27) respondents, while Ou-Nick Health Centre accounted for 3.7% (n = 11). Most respondents had 1 to 5 years of work experience (46.4%, n = 137), while fewer had extensive work experience of 16 or more years (13.6%, n = 40). The majority of respondents were registered nurses (63.1%, n = 186), with the remainder (36.9%, n = 109) being enrolled nurses. Registered nurses (63.1%) were the most affected by occupational injuries among different nursing categories. Table 1 presents a summary of the demographic characteristics.

### 3.2. Prevalence of Occupational Injuries Among Nurses

**Figure 2:**
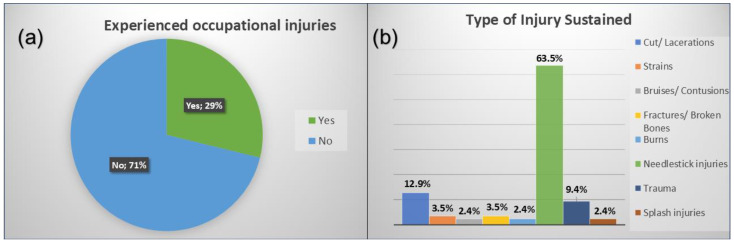
(**a**,**b**). Respondents’ injury experience and types of occupational injuries among respondents.

Among the 295 participating nurses, 28.8% (n = 85) reported experiencing at least one occupational injury in the past 12 months (Figure 2a), with a 95% confidence interval (CI) of 24.3–33.3%. This corresponds to an annual incidence rate of 288 occupational injuries per 1000 exposed nurses. 

### 3.3. Types and Causes of Injuries

As presented in Figure 2b, NSIs were the most prevalent type of occupational injury among nurses (63.5%), followed by cuts and lacerations (12.9%). The primary causes of these injuries were accidental needle pricks (63.5%), sharp instrument injuries (10.6%), and slip, trip, or fall accidents (8.2%). The most commonly affected body parts were the upper extremities, including hands and arms (72.9%). The highest injury rates were recorded in the maternity ward (17.6%) and surgical ward (16.5%), where exposure to sharp instruments and high patient turnover may have contributed to the increased risk.

### 3.4. Department Where Injury Occurred

**Figure 3:**
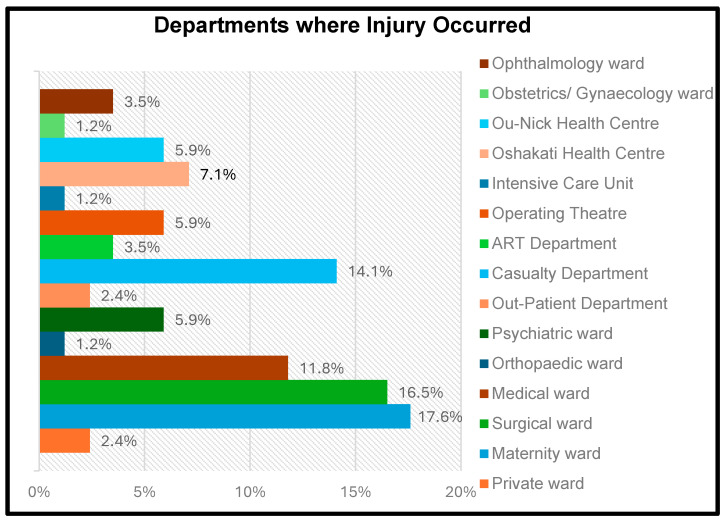
Distribution of injuries by department.

As shown in Figure 3, the maternity ward emerged as the most frequently reported location where occupational injuries occurred among nurses, accounting for 17.6% of injuries. This was followed closely by the surgical ward (16.5%) and the casualty department (14.1%). These departments are typically characterised by high patient turnover, increased physical demands, and exposure to sharp instruments, which may increase the risk of occupational injuries.

### 3.5. Reporting and Medical Attention for Injured Nurses

Most occupational injuries (87.1%) occurred in hospital settings, highlighting the high-risk nature of these environments. A significant proportion of injured nurses (11.8%) did not report their injuries to management or relevant authorities. In addition, 10.6% of injured nurses did not seek medical attention. The majority of respondents (29.8%) rated the support post-injury as “excellent”, suggesting that, in some instances, nurses received exceptional and timely assistance following an injury.

### 3.6. Descriptive Statistics for Secondary Data

All nurses (100%; n = 7) who experienced occupational injuries were female (see Table 2). The most affected age group was 36–45 years, accounting for 42.9% of cases. Most injured nurses (85.7%; n = 6) were registered nurses, indicating they may have had greater exposure to tasks involving potential injury risks. The predominant type of injury was needlestick injuries, constituting 71.4% of reported cases. These findings aligned with results from the analysis of primary data.

### 3.7. Contributing Factors from Secondary Data

The occupational injury register data identify key contributors to occupational injuries. Poor workplace organisation emerged as the most frequently reported factor, accounting for 57.1% of cases. Work-related stress and an unsafe work environment were each cited in 28.6% of cases, while inadequate training and education were noted in 14.3% of cases. However, factors such as work overload, inadequate staffing, poor shift patterns, and lack of safety equipment were not reported. The findings depend on the register’s accuracy and completeness, which may influence the overall insights.

### 3.8. Tests for Normality

The Kolmogorov–Smirnov test was applied to demographic data to assess the normality of the primary data. The null hypothesis (H_0_) assumed a normal distribution, while the alternative hypothesis (H_1_) suggested that the data were not normally distributed. As illustrated in Table 3, the results showed that the *p*-values for all the demographic factors were less than 0.05, indicating that the data did not follow a normal distribution. Consequently, non-parametric statistical methods were used for analysis.

### 3.9. Chi-Square Test Analysis

The Pearson chi-square test was applied to assess the association between demographic factors (such as age, sex, work experience) and the dependent variable (occupational injuries). As presented in Table 4, the findings indicated no significant association with place of work (*p* = 0.083), sex (*p* = 0.990), and age (*p* = 0.114). However, significant associations were observed for the highest level of education (*p* = 0.027), years of experience (*p* = 0.029), and employment status (*p* = 0.012). The assessment of the association between contributing factors and occupational injuries revealed that lack of proper training and education (*p* < 0.001), work overload (*p* < 0.001), inadequate staffing levels (*p* = 0.009), poor shift patterns (*p* = 0.016), insufficient safety equipment (*p* = 0.004), and poor work organisation (*p* = 0.011) were significantly associated with injuries. Conversely, work-related stress (*p* = 0.201) and an unsafe work environment (*p* = 0.158) were not significantly linked to injury prevalence.

### 3.10. Univariate and Multivariate Logistic Regression

Univariate logistic regression was used to further explore the individual effect of each independent variable on the outcome, providing odds ratios and confidence intervals. It modelled the probability of experiencing an occupational injury (yes/no) based on predictor variables, specifically demographic data and contributing factors. A significance threshold (*p*-value) of 0.25 was applied in this analysis to avoid excluding relevant predictors. This threshold was selected based on established statistical practice, where a more inclusive criterion in the bivariate stage is recommended to avoid excluding potentially important predictors prematurely.

Independent variables with a *p*-value ≤ 0.25 were included in the multivariate logistic regression model, allowing for the further exploration of their effect on the outcome while controlling for potential confounders. Univariate logistic regression (Table 5) demonstrated that higher levels of education increased injury likelihood, with certificate/diploma holders being 2.9 times more likely (COR = 2.90; 95% CI: 1.22–6.88, *p* = 0.016) and bachelor’s degree holders being 3.08 times more likely (COR = 3.08; 95% CI: 1.28–7.44, *p* = 0.012) to be at a higher risk. Nurses with 1–5 years of experience were 2.75 times more likely to sustain injuries (COR = 2.75; 95% CI: 1.30–5.82, *p* = 0.008). Employment status as a registered nurse was protective, reducing the likelihood of injuries by 51% (COR = 0.49; 95% CI: 0.28–0.86, *p* = 0.013). Key predictors of injury included inadequate staffing levels (COR = 2.37; 95% CI: 1.23–4.59, *p* = 0.010), poor shift patterns (COR = 2.91; 95% CI: 1.18–7.16, *p* = 0.020), inadequate safety equipment (COR = 2.12; 95% CI: 1.26–3.59, *p* = 0.005), and poor workplace organisation (COR = 2.24; 95% CI: 1.19–4.19, *p* = 0.012). The lack of proper training and education significantly increased the likelihood of injuries, with nurses in this category being 3.64 times more likely to report injuries (COR = 3.64; 95% CI: 1.96–6.77, *p* = 0.001).

The multivariate logistic regression analysis was then performed to adjust for potential confounding variables and determine which factors were independent predictors of occupational injuries among nurses. It identified several key factors influencing occupational injuries among nurses (Table 5). Among the significant predictors, education level, employment status, and inadequate training emerged as critical determinants of injury risk. Specifically, nurses holding a bachelor’s degree were found to be 3.30 times more likely to sustain occupational injuries compared to those with lower educational qualifications (AOR = 3.30, 95% CI: 1.11–9.81, *p* = 0.03). In addition, nurses who had not received adequate training and education had 3.27 times higher odds of experiencing occupational injuries (AOR = 3.27, 95% CI: 1.62–6.61, *p* < 0.001), highlighting the strong association between insufficient training and increased injury prevalence. Employment as a registered nurse was a protective factor, reducing the likelihood of occupational injuries by 70% (AOR = 0.30, 95% CI: 0.12–0.74, *p* = 0.01). After adjusting for other variables, registered nurses are less likely to be injured compared to enrolled nurses. Other assessed variables, including years of experience, workplace safety conditions, staffing levels, shift patterns, availability of safety equipment, work-related stress, and poor workplace organisation, did not show statistically significant associations with injury risk.

### 3.11. Comparison of Primary and Secondary Data

The comparison of primary and secondary data revealed both similarities and differences in the prevalence, nature, affected body parts, and causes of occupational injuries among nurses. Needlestick injuries were the most frequently reported, with secondary data indicating a prevalence of 71.4% and primary data indicating a prevalence of 63.5%, highlighting their significance in clinical practice. Both datasets identified the upper extremities as the most commonly injured. However, in the secondary data, 42.9% was recorded, and in the primary data, which included injuries to the shoulders, arms, and hands, a higher prevalence of 72.9% was reported. Injuries predominantly occurred in high-risk departments, with secondary data pointing to the surgical ward (28.6%) and primary data highlighting maternity (17.6%), surgical (16.5%), and casualty (14.1) wards. Contributing factors included environmental and organisational challenges; in the secondary data, poor workplace organisation was cited (57.1%), while in the primary data, injuries were attributed to work overload (66.8%), unsafe work environment (46.4%), inadequate safety equipment (47.1%), and lack of training (36.3%). Both datasets indicated that most injuries were minor, with affected nurses typically returning to work immediately.

## 4. Discussion

### 4.1. Prevalence of Occupational Injuries

Findings from this study indicate that 28.8% of nurses experienced at least one occupational injury over the past 12 months, equating to an annual incidence rate of 288 injuries per 1000 nurses. These results correspond with prior research [6,11], which reported prevalence rates ranging from 29.7% to 60.2% in low- and middle-income countries (LMICs). For example, a related investigation in Saudi Arabia found a higher prevalence of occupational injuries at 52% among 387 healthcare workers [29]. The comparability of these findings is notable, as these studies were conducted in different healthcare environments that nevertheless share common challenges, such as limited resources and workforce constraints.

However, this study advances current understanding by providing context-specific evidence from Namibia, where limited empirical data exist on occupational injuries among nurses. In contrast to broader studies like that of Debelu et al. [10], which reported a higher average prevalence of 87.8% across various healthcare workers in LMICs, our study focuses exclusively on nurses, a critical yet often understudied group in the occupational health literature. This targeted focus allows for more precise insights into profession-specific risks and implications.

The differences in prevalence rates may reflect distinct contextual factors, such as variations in safety regulations, institutional reporting practices, and healthcare infrastructure. Namibia’s healthcare system, for instance, may have different protocols or enforcement mechanisms related to occupational safety, which could influence injury reporting and prevention.

Although the prevalence observed in this study is lower than some estimates reported for sub-Saharan Africa, it still constitutes a significant public health concern. The finding that nearly one-third of nurses sustained occupational injuries emphasises the need for evidence-based policy interventions to improve workplace safety in Namibia’s public health facilities. By highlighting the scope of this issue in a previously under-researched setting, this study not only confirms global trends but also fills a regional knowledge gap and informs localised occupational health strategies.

These findings align with broader patterns observed in LMICs, where resource limitations and inadequate occupational health and safety (OHS) frameworks contribute to higher injury rates [10]. In contrast, countries with well-established OHS systems report significantly lower prevalence rates [17], suggesting that structural improvements in policy and practice could yield substantial health and safety benefits for frontline healthcare workers in Namibia. Evidence suggests that adopting safety interventions in high-income countries has significantly reduced injury rates [8]. Efforts by the Namibian government, particularly through the Ministry of Labour, Industrial Relations, and Employment Creation, to develop legislation to improve workplace safety and health [30], represent a positive step towards addressing these challenges.

### 4.2. Types of Injuries

Needlestick injuries emerged as the most frequently reported occupational injury, accounting for 63.5% of cases. These findings are consistent with a Namibian cross-sectional study by Moyo et al. [7], which recorded a 12-month prevalence of needlestick injuries at 27.4% among 400 healthcare workers in a private hospital in Oshakati. This outcome aligns with the existing literature, identifying needlestick injuries as a significant occupational hazard for healthcare personnel, particularly nurses [2,12]. The prevalence observed in this study reflects frequent exposure to accidental needle pricks, inadequate compliance with safety protocols, and limited training on safe handling practices. These challenges are further compounded by resource constraints and staffing shortages, which can increase workloads and reduce compliance with standard precautions.

Research conducted in Ghana by Appiagyei et al. [11] reported a lower prevalence (35.4%) of needlestick injuries among healthcare professionals. Similarly, a prevalence of 23% was found in Nigeria among doctors, nurses, and laboratory technicians [31], suggesting that a greater awareness of safe injection practices may have contributed to the reduced incidence in these studies. However, it is important to note that the study population in these studies included health professionals in general, rather than focusing exclusively on nurses.

The risk associated with needlestick injuries extends to the exposure to bloodborne pathogens such as the Hepatitis B virus (HBV), Hepatitis C virus (HCV), and HIV, further emphasising their severity. Research by Rai et al. [8] emphasises the importance of HBV vaccination and timely post-exposure prophylaxis (PEP) for HIV. Nevertheless, the uptake of these protective measures remains inconsistent, as demonstrated by a study in Ethiopia where access to vaccinations and PEP was irregular [16]. These results highlight the necessity of targeted training initiatives and the stricter enforcement of vaccination and PEP protocols.

Cuts and lacerations were the second most prevalent injury type, comprising 12.9% of cases. These injuries typically result from handling sharp objects without sufficient protective measures or training. A South African study by Denge and Rakhudu [14] found that 14.3% of nurses sustained cuts and lacerations due to inadequate personal protective equipment (PPE) and insufficient safety procedures. Conversely, Mossburg et al. [17] reported a lower prevalence (8.7%) in the United Kingdom, attributing this reduction to more stringent occupational safety regulations.

Less commonly reported injuries included bruising/contusions, burns, and splash injuries, each constituting 2.4% of cases. Similar trends have been documented in the literature. Denge and Rakhudu [14] identified that contusions, burns, and splash injuries collectively represented approximately 3% of occupational injuries among South African nurses, closely aligning with the current findings. Research by Appiagyei et al. [11] in Ghana also found that splash injuries, including those resulting from exposure to hazardous chemicals and bodily fluids, accounted for 2.7% of reported cases. Given that the study sample included nurses employed in public healthcare facilities, the demographic and workplace conditions were comparable to those in the present study. The relatively low prevalence of splash injuries may be linked to improved adherence to PPE usage and enhanced safety policies.

### 4.3. Causes of Injuries

Accidental needlestick injuries were identified as the most common cause of occupational injuries, accounting for 63.5% of cases. These findings align with global research on nurses’ susceptibility to sharps-related injuries [8,12]. Such injuries carry significant risks, including exposure to bloodborne infections like HBV, HCV, and HIV. Research suggests these risks are exacerbated by insufficient vaccination coverage and limited PEP utilisation [8]. The high prevalence of needlestick injuries observed in this study is consistent with trends reported worldwide [6,12], particularly in clinical settings where surgical procedures increase the likelihood of such incidents. A study by Erturk Sengel et al. [12] in Turkey found that needlestick injuries accounted for 60% of occupational injuries among nurses, closely mirroring the present study’s findings. The comparability of the sample populations, which included nurses from high-risk hospital wards, strengthens this comparison. In contrast, research by Hawkins and Ghaziri [6] conducted in a high-income healthcare setting in the United States reported a lower prevalence of needlestick injuries (45%), likely due to improved adherence to occupational safety protocols, the widespread availability of safety-engineered devices, and enhanced training.

Sharp instrument or equipment injuries and exposure to blood or bodily fluids each comprised 10.6% of cases. These findings are consistent with research documenting similar risks in resource-limited healthcare settings [11]. Slip, trip, and fall incidents accounted for 8.2% of cases, aligning with observations by Kim and Jeong [32], who identified environmental hazards and physical strain as key contributors to injury rates in healthcare settings. Their study, conducted in South Korea, attributed these incidents to factors such as wet floors, improper footwear, and physical strain, which are also relevant in this study. Additional causes of injuries included violence, contact with hazardous chemicals, and equipment malfunction, each accounting for 2.4% of cases. Workplace violence findings align with Hawkins and Ghaziri [6], who observed a rising trend in intentional occupational injuries among healthcare workers in the United States. Similarly, Courtice, Olsson, and Cherrie [33] highlighted that insufficient PPE and improper chemical handling protocols contribute to injuries related to hazardous substance exposure. Equipment malfunction was also identified as a contributing factor, reinforcing the need for continuous training in the proper use of medical equipment.

### 4.4. Anatomical Sites Affected by Injuries

This study indicated that injuries to the upper extremities were the most commonly reported, comprising 72.9% of cases. This finding corresponds with research conducted in South Africa and Malaysia, which identified these areas as particularly susceptible due to repetitive movements, patient handling, and the frequent use of medical instruments [14,34]. Injuries to the lower extremities (10.6%) and skin (5.9%) further emphasise the physical demands of nursing and exposure to hazardous environments. The back/spine, torso, and eyes exhibited a lower injury prevalence (1.2% each), potentially indicating the effectiveness of protective measures and reduced exposure.The findings identified the maternity ward as the department with the highest frequency of occupational injuries (17.6%), followed by the surgical ward (16.5%) and the casualty department (14.1%). Conversely, the orthopaedic ward, intensive care unit (ICU), and obstetrics/gynaecology ward had lower injury rates, representing 1.2% of cases. These results align with Denge and Rakhudu [14], who highlighted that high-risk departments, such as maternity and surgical wards, are often associated with physically demanding tasks, uncooperative patients, and the frequent handling of sharp instruments, all of which contribute to a heightened risk of injury.

### 4.5. Distribution of Injuries Among Hospital Departments

The elevated prevalence of occupational injuries in the maternity ward aligns with Mossburg et al. [17], who found that maternal care settings present increased risks due to frequent patient handling, emergency deliveries, and exposure to bodily fluids. Similarly, Debelu et al. [10] reported high injury rates in maternity wards due to the physically intensive nature of obstetric care, which includes lifting, repositioning patients, and handling sharp instruments. Rai et al. [8] also identified a high prevalence of sharp injuries in surgical units due to the frequent use of scalpels, suturing needles, and other sharp instruments. Surgical nurses are subjected to prolonged standing, high workloads, and fast-paced work environments, all of which contribute to occupational injuries. Hawkins and Ghaziri [6] emphasised the high risk of injuries in emergency and casualty departments due to the unpredictable nature of cases, exposure to violence, and the necessity for urgent decision-making under pressure.

On the other hand, the lower injury rates in the orthopaedic ward, ICU, and obstetrics/gynaecology ward may be attributed to differences in workload, patient mobility, and procedural risks. Despite operating in high-risk environments, Denge and Rakhudu [14] observed that ICU nurses benefit from structured workflows, stringent safety protocols, and greater access to protective equipment, which may reduce injury risks. Likewise, Ahn et al. [35] found that obstetrics and gynaecology nurses experience fewer occupational injuries, as their work is less physically demanding.

Globally, occupational injury distribution varies across departments based on healthcare system structures, staffing levels, and institutional safety measures. In Ethiopia, Debelu et al. [10] identified casualty departments as having the highest injury rates, in contrast to this study, where the maternity ward reported the most injuries. These variations may result from differences in patient load and resource allocation. Similarly, a South Korean study by Kim and Jeong [32] found that surgical units accounted for the highest number of occupational injuries, with needlestick injuries being the most prevalent. While this partially aligns with the present study, differences in injury distribution may stem from variations in safety training, PPE availability, and adherence to occupational health policies.

### 4.6. Comparison of Injuries Among Nurses Working in Different Health Facilities

In the hospital setting, the proportion of injuries (87%) closely mirrored the percentage of respondents (87.1%), reflecting a consistent risk distribution across departments. At Oshakati Health Centre, the injury rate (7.1%) was slightly lower than the proportion of respondents (9.2%), suggesting a comparatively safer work environment. Conversely, Ou-Nick Health Centre exhibited a disproportionately higher injury rate (5.9%) relative to its respondent proportion (3.7%), potentially due to limited safety resources or a heightened exposure to occupational hazards. This observation is consistent with Appiagyei et al. [11], who found that facilities with resource constraints and inadequate safety measures tend to report higher injury rates, as demonstrated in their study in Ghana.

### 4.7. Reporting of Injuries to Authorities

This study revealed that 87.1% of nurses in the Oshakati District reported occupational injuries to relevant authorities, whereas 11.8% did not, and 1.2% were uncertain. These findings are consistent with global trends indicating high nurse reporting rates [10,14]. The high reporting rate suggests an increased awareness of workplace safety protocols and the effectiveness of standardised systems, with most nurses acknowledging the importance of reporting injuries to access medical care, per Namibia’s Labour Act, 2007 [36]. However, 11.8% of non-reporting participants likely faced barriers similar to those identified in the literature, such as a lack of protocol awareness [9,17].

The low reporting rates to the District Infection Control Department at Oshakati Hospital highlight a significant concern. While injuries are reported to authorities, documentation remains minimal, possibly due to the absence of occupational injury registers in the health facilities which were under study. This finding aligns with Amuele [36], who reported that institutional limitations and the absence of designated personnel to facilitate reporting contribute to underreporting. Similarly, underreporting to the Social Security Commission may be due to unfamiliar reporting procedures and insufficient guidance. This observation mirrors Nadalin and Smith [9], who found that 64% of healthcare workers in Canada failed to report workplace injuries due to limited knowledge. In contrast, López Gómez et al. [37] found that underreporting was less prevalent in Spain due to structured reporting mechanisms and strong legal enforcement, suggesting that well-established occupational health frameworks significantly influence injury reporting rates.

### 4.8. Medical Care Accessed Post-Injury

A significant majority (89.4%) of participants who experienced occupational injuries sought medical attention, while 10.6% did not. This highlights the importance nurses place on their health when injured and emphasises the necessity of a robust healthcare system that provides timely care for healthcare workers. Debelu et al. [10] found that in developing countries, 72% to 90% of healthcare workers sought medical treatment post-injury, depending on setting and injury type. In contrast, Nadalin and Smith [9] reported that 64% of injured healthcare workers in Canada neither formally reported their injuries nor sought medical attention, often due to the fear of repercussions, underestimation of severity, or time constraints.

### 4.9. Severity of Injuries

This study found that most nurses (62.4%) sustained minor injuries requiring minimal medical treatment, while 36.5% experienced moderate injuries necessitating medical care but not hospitalisation. Only 1.2% reported life-threatening injuries. These findings are consistent with previous research, which indicates that minor and moderate injuries are the most common among healthcare workers. Life-threatening injuries remain rare [12]. The majority (64.7%) resumed work immediately after sustaining an injury, a trend consistent with studies suggesting that nurses often return to work quickly due to institutional pressures and the underestimation of injury severity [10]. However, a small percentage (2.4%) required more than a week to return, potentially indicating more severe injuries or barriers to healthcare access [11]. A considerable proportion (29.8%) rated post-injury support as “excellent”, while 38.6% found it “good”. However, 6.8% rated support as “very poor”, suggesting potential deficiencies in medical attention and follow-up care [9].

### 4.10. Factors Contributing to Occupational Injuries

The Pearson chi-square test identified significant relationships between various factors and the prevalence of occupational injuries among nurses, highlighting the necessity for targeted interventions. Education level (*p* = 0.027) and years of experience (*p* = 0.029) were significantly associated with injury rates. Higher education was associated with a lower risk of injury, as nurses with advanced education are more proficient in managing clinical risks [38]. While extensive experience can offer protective benefits, prolonged exposure to demanding work environments may result in complacency and burnout, thereby increasing injury susceptibility [39]. Employment status (*p* = 0.012) also played a critical role in injury risk. The finding that registered nurses had a 70% lower likelihood of experiencing occupational injuries aligns with previous research, highlighting the impact of professional training and advanced skill sets in minimising workplace hazards [38]. Registered nurses undergo extensive education and training, equipping them to handle complex clinical scenarios safely. Conversely, enrolled nurses may lack the training to navigate hazardous situations effectively.

A key finding was the significant association between the lack of proper training and education and occupational injuries (*p* = 0.001), which was closely linked to an increased risk of injury. The lack of training on safety procedures was a major factor contributing to injuries [12]. Understaffing (*p* = 0.009) and irregular shift patterns (*p* = 0.016) were also identified as significant contributors, reinforcing prior studies that associate inadequate staffing and inconsistent shifts with fatigue, stress, and errors [40]. In addition, insufficient staffing intensifies both the physical workload and mental strain, while irregular shifts disrupt circadian rhythms, heightening vulnerability to injuries [5]. The lack of adequate safety equipment (*p* = 0.004) was another significant factor, emphasising the critical role of proper protective equipment in injury prevention. Ensuring that healthcare facilities are well-equipped and that nurses receive adequate training on the use of safety equipment is essential. Furthermore, disorganised work environments (*p* = 0.011) were significantly linked to injuries, suggesting that inefficient workflows, poor communication, and insufficient management support create unsafe working conditions [1].

The findings from the univariate and multivariate logistic regression analyses identified significant predictors of occupational injuries among nurses. Notably, nurses holding a bachelor’s degree exhibited 3.30 times higher odds of sustaining occupational injuries (AOR = 3.30, 95% CI: 1.11–9.81, *p* = 0.03). This may be attributed to a more frequent exposure to complex clinical tasks and higher workloads typically assigned to degree-holding nurses, which could increase their injury risk. Inadequate training and education increased the likelihood of occupational injuries by 3.27 times (AOR = 3.27, 95% CI: 1.62–6.61, *p* < 0.001). Conversely, employment as a registered nurse demonstrated a protective effect, reducing the odds of sustaining occupational injuries by 70% (AOR = 0.30, 95% CI: 0.12–0.74, *p* = 0.01). These findings align with the existing literature, which emphasises the role of education, training, and employment status in influencing occupational injury prevalence [10,18,38].

Education level plays a crucial role in occupational injury risk. While advanced education provides nurses with critical skills for managing complex situations, it may also expose them to increased workplace demands, leading to higher injury risks [38]. Similarly, nurses with insufficient training face difficulties in safely managing occupational hazards, increasing their susceptibility to injuries [11,41]. Regular training in personal protective equipment (PPE) use, the safe handling of medical devices, and emergency protocols is essential in mitigating these risks [12]. However, if training programs are outdated or inadequately implemented, their effectiveness in reducing injuries remains limited [14].

The protective effect observed among registered nurses may be attributed to their extensive training and experience in handling occupational hazards. Registered nurses are typically more familiar with safety protocols and possess greater competency in high-risk environments, reducing their likelihood of sustaining injuries [18]. Conversely, enrolled nurses, who often perform routine tasks with less autonomy, may lack the necessary training to avoid hazardous situations, making them more vulnerable to workplace injuries [14].

## 5. Study Limitations

The lack of occupational injury registers in certain public health facilities in the Oshakati District posed challenges for data abstraction, as self-reported information could not be cross-verified with official records. This limitation affected this study’s ability to detect inconsistencies and assess response reliability. To mitigate this, data were sourced from the District Infection Control Department. The response bias associated with self-reported data was a potential limitation of this study. Participants may have underreported or overreported occupational injuries due to recall challenges or social desirability. To mitigate this, the questionnaire was carefully designed to ensure clarity and anonymity, encouraging honest responses. In addition, participants were assured of confidentiality and informed that the data would be used solely for research purposes.

## 6. Conclusions

The reported prevalence rate of 28.8% confirmed that occupational injuries are a significant concern, supporting the study hypothesis that such injuries are common among nurses in Oshakati District public health facilities. The observed significant associations between injury prevalence and workplace factors highlighted the influence of these workplace conditions on occupational injuries. Enhancing the workplace environment by mitigating these factors is essential to reducing occupational injuries and improving nurse safety and well-being. Practical measures such as regular and relevant safety training and updating and enforcing occupational health and safety laws will contribute to safer working conditions and improve the Namibian health system and healthcare service delivery. While this study primarily relied on self-reported data, limited external validation was achieved through the abstraction of seven records from occupational injury registers, supporting the credibility of some reported incidents. Future research should explore the role of nursing errors in occupational injuries to provide a more comprehensive understanding of their impact. It should also assess the specific causes of underreporting and poor documentation of occupational injuries in the Oshakati District.

To systematically address occupational health and safety concerns in the Oshakati District, it is recommended that occupational health departments be established at the Intermediate Hospital Oshakati and primary healthcare facilities. This should include allocating dedicated occupational health personnel, such as occupational health nurses and safety officers, and investing in appropriate infrastructure and equipment to support these services. To improve and standardise injury reporting and documentation in the Oshakati District, occupational injury registers containing incident reporting forms should be available and utilised across all hospital departments and primary healthcare facilities. More focused attention should be rendered to address mental health challenges resulting from occupational injuries, such as stress, anxiety, and psychological trauma, in the Oshakati District. This may include establishing staff counselling programs, offering regular mental health screenings, and integrating psychological support into routine occupational health services. Furthermore, the ongoing surveillance of occupational injuries, supported by regular data analysis and review, will facilitate trend monitoring and support evidence-based policy development and resource allocation to enhance workplace safety in the Oshakati District.

## Figures and Tables

**Table 1:** Demographic characteristics of nurses (n = 295).

Variable	Demographic Factors	Frequency (n)	Percentage (%)
Sex	Male	45	15.3
	Female	250	84.7
Age	18–25	39	13.2
	26–35	128	43.4
	36–45	84	28.5
	46–55	28	9.5
	56+	16	5.4
Highest educational level	Certificate/diploma	147	49.8
	Bachelor’s degree	123	41.7
	Postgraduate degree	25	8.5
Place of work	Private ward	6	2.0
	Maternity ward	61	20.7
	Surgical ward	30	10.2
	Orthopaedic ward	22	7.5
	Psychiatric ward	9	3.1
	Outpatient department (OPD)	29	9.8
	Emergency department (casualty)	19	6.4
	Paediatric medical ward	4	1.4
	X-ray department	2	0.7
	ART department	9	3.1
	Operating theatre	15	5.1
	Intensive care unit (ICU)	10	3.4
	Central Sterile Supply Department (CSSD)	2	0.7
	Oshakati Health Centre	27	9.2
	Ou-Nick Health Centre	11	3.7
	Paediatric surgical ward	7	2.4
	TB ward	3	1.0
	Ophthalmology ward	5	1.7
	Oncology	6	2.0
	Dialysis ward	1	0.3
Years of experience	1–5 years	137	46.4
	6–10 years	83	28.1
	11–15 years	35	11.9
	16 years or more	40	13.6
Employment status	Registered nurse	186	63.1
	Enrolled nurse	109	36.9

**Table 2:** Demographic factors for secondary data (n = 7).

Variable	Demographic Factors	Frequency (n)	Percentage (%)
Sex	Female	7	100
Age	18–25	1	14.3
	26–35	2	28.6
	36–45	3	42.9
	46–55	0	0
	56+	1	14.3
Employment status	Registered nurse	6	85.7
	Enrolled nurse	1	14.3

**Table 3:** Kolmogorov–Smirnov test of normality for demographic data.

Factor	*p*-Value	Significance
Place of work	<0.001	Significant; Reject null hypothesis
Sex	<0.001	Significant; Reject null hypothesis
Age	<0.001	Significant; Reject null hypothesis
Highest level of education	<0.001	Significant; Reject null hypothesis
Years of experience	<0.001	Significant; Reject null hypothesis
Employment status	<0.001	Significant; Reject null hypothesis

**Table 4:** Chi-square test for significance of demographic and contributing factors.

Factor	Pearson Chi-Square (*p*-Value)	Significance
Demographic factors		
Place of work	0.083	No significant association
Sex	0.990	No significant association
Age	0.114	No significant association
Highest level of education	0.027	Significant association
Years of experience	0.029	Significant association
Employment status	0.012	Significant association
Contributing factors		
Unsafe work environment	0.158	No significant association
Work overload	0.630	No significant association
Lack of proper training and education	0.001	Significant association
Inadequate staffing levels	0.009	Significant association
Poor shift patterns	0.016	Significant association
Inadequate safety equipment	0.004	Significant association
Work-related stress	0.201	No significant association
Poor work organisation	0.011	Significant association

**Table 5:** Crude and adjusted odds ratios for occupational injury prevalence among nurses by demographic and contributing factors.

Variable	Univariate Logistic Regression			Multivariate Logistic Regression		
	*p*-Value	Crude Odds Ratio	Confidence Interval (95%)	*p*-Value	Adjusted Odds Ratio	Confidence Interval (95%)
Demographic characteristics						
Highest level of education (certificate/diploma)	0.02	2.90	1.22–6.88	0.80	1.16	0.38–3.51
Highest level of education (bachelor’s degree)	0.01	3.08	1.28–7.44	0.03	3.30	1.11–9.81
Years of experience (1–5 years)	0.01	2.75	1.30–5.82	0.48	1.40	0.54–3.63
Years of experience (6–10 years)	0.23	1.62	0.74–3.53	0.62	0.79	0.30–2.04
Employment status (registered nurses)	0.01	0.49	0.28–0.86	0.01	0.30	0.12–0.74
Contributing factors						
Unsafe work environment	0.16	1.44	0.87–2.41	0.74	0.90	0.49–1.65
Lack of proper training and education	0.01	3.64	1.96–6.77	0.01	3.27	1.62–6.61
Inadequate staffing levels	0.01	2.37	1.23–4.59	0.12	1.89	0.85–4.19
Poor shift patterns	0.02	2.91	1.18–7.16	0.55	1.38	0.48–3.99
Inadequate safety equipment	0.01	2.12	1.26–3.59	0.08	1.71	0.93–3.15
Work-related stress	0.20	1.44	0.82–2.51	0.76	0.90	0.45–1.79
Poor workplace organisation	0.01	2.24	1.19–4.19	0.89	1.06	0.49–2.28

## Data Availability

The data presented in this study are available on request from the corresponding author.
